# Acute hydrocortisone administration reduces cardiovagal baroreflex sensitivity and heart rate variability in young men

**DOI:** 10.1113/JP276644

**Published:** 2018-09-12

**Authors:** Ahmed M. Adlan, Jet J. C. S. Veldhuijzen van Zanten, Gregory Y. H. Lip, Julian F. R. Paton, George D. Kitas, James P. Fisher

**Affiliations:** ^1^ College of Life and Environmental Sciences University of Birmingham Edgbaston Birmingham UK; ^2^ University of Birmingham Institute of Cardiovascular Sciences City Hospital Birmingham UK; ^3^ Faculty of Medical and Health Sciences University of Auckland Auckland New Zealand; ^4^ Department of Rheumatology Dudley Group NHS Foundation Trust Russells Hall Hospital Dudley West Midlands UK

**Keywords:** Cortisol, glucocorticoid, autonomic nervous system, stress

## Abstract

**Key points:**

- A surge in cortisol during acute physiological and pathophysiological stress may precipitate ventricular arrhythmia and myocardial infarction.
- Reduced cardiovagal baroreflex sensitivity and heart rate variability are observed during acute stress and are associated with an increased risk of acute cardiac events.
- In the present study, healthy young men received either a single iv bolus of saline (placebo) or hydrocortisone, 1 week apart, in accordance with a randomized, placebo‐controlled, cross‐over study design.
- Hydrocortisone acutely increased heart rate and blood pressure and reduced cardiovagal baroreflex sensitivity and heart rate variability in young men.
- These findings suggest that, by reducing cardiovagal baroreflex sensitivity and heart rate variability, acute surges in cortisol facilitate a pro‐arrhythmic milieu and provide an important mechanistic link between stress and acute cardiac events

**Abstract:**

Surges in cortisol concentration during acute stress may increase cardiovascular risk. To better understand the interactions between cortisol and the autonomic nervous system, we determined the acute effects of hydrocortisone administration on cardiovagal baroreflex sensitivity (BRS), heart rate variability (HRV) and cardiovascular reactivity. In a randomized, placebo‐controlled, single‐blinded cross‐over study, 10 healthy males received either a single iv bolus of saline (placebo) or 200 mg of hydrocortisone, 1 week apart. Heart rate (HR), blood pressure (BP) and limb blood flow were monitored 3 h later, at rest and during the sequential infusion of sodium nitroprusside and phenylephrine (modified Oxford Technique), a cold pressor test and a mental arithmetic stress task. HRV was assessed using the square root of the mean of the sum of the squares of differences between successive R‐R intervals (rMSSD). Hydrocortisone markedly increased serum cortisol 3 h following infusion and also compared to placebo. In addition, hydrocortisone elevated resting HR (+7 ± 4 beats min^−1^; *P* < 0.001) and systolic BP (+5 ± 5 mmHg; *P* = 0.008); lowered cardiovagal BRS [geometric mean (95% confidence interval) 15.6 (11.1–22.1) ms/mmHg *vs*. 26.2 (17.4––39.5) ms/mmHg, *P* = 0.011] and HRV (rMSSD 59 ± 29 ms *vs*. 84 ± 38 ms, *P* = 0.004) and increased leg vasoconstrictor responses to cold pressor test (Δ leg vascular conductance −45 ± 20% *vs*. −23 ± 26%; *P* = 0.023). In young men, an acute cortisol surge is accompanied by increases in HR and BP, as well as reductions in cardiovagal BRS and HRV, potentially providing a pro‐arrhythmic milieu that may precipitate ventricular arrhythmia or myocardial infarction and increase cardiovascular risk.

## Key points

- A surge in cortisol during acute physiological and pathophysiological stress may precipitate ventricular arrhythmia and myocardial infarction.
- Reduced cardiovagal baroreflex sensitivity and heart rate variability are observed during acute stress and are associated with an increased risk of acute cardiac events.
- In the present study, healthy young men received either a single iv bolus of saline (placebo) or hydrocortisone, 1 week apart, in accordance with a randomized, placebo‐controlled, cross‐over study design.
- Hydrocortisone acutely increased heart rate and blood pressure and reduced cardiovagal baroreflex sensitivity and heart rate variability in young men.
- These findings suggest that, by reducing cardiovagal baroreflex sensitivity and heart rate variability, acute surges in cortisol facilitate a pro‐arrhythmic milieu and provide an important mechanistic link between stress and acute cardiac events

## Introduction

Cortisol is the principal glucocorticoid in humans and has important metabolic (e.g. mobilization of glucose, fatty acids and amino acids), immune (e.g. anti‐inflammatory) and cardiovascular (e.g. maintenance of normal blood pressure) functions. The hypothalamic‐pituitary‐adrenal (HPA) axis is activated during periods of stress and stimulates the production and release of cortisol. The surge in cortisol during acute stress may precipitate left ventricular dysfunction, ventricular arrhythmia and myocardial infarction (Brotman *et al*. 2007). Although the interaction between cortisol and the autonomic nervous system has been implicated in the increased cardiovascular risk of acute stress (Brotman *et al*. 2007), the modulatory effects of cortisol on cardiac autonomic control remain incompletely understood.

Reduced cardiovagal baroreflex sensitivity (BRS) has been identified in a variety of cardiovascular diseases (Eckberg & Sleight, 1992) and has been shown to predict mortality following myocardial infarction (La Rovere *et al*. 1998) and in patients with chronic heart failure (Mirizzi *et al*. 2013). Acute mental stress impairs BRS (Broadley *et al*. 2005; Durocher *et al*. 2011), potentially facilitating a pro‐arrhythmic milieu and providing an important mechanistic link between mental stress and acute cardiac events (Finlay *et al*. 2016). In healthy humans, exogenous administration of cortisol acutely (within 6 h) increases heart rate (HR) (Dodt *et al*. 2000; Heindl *et al*. 2006) via a number of suggested mechanisms, including parasympathetic withdrawal and reduced cardiovagal BRS. Although experimental evidence is presently lacking in humans, evidence from animal studies supports the hypothesis that glucocorticoids can elicit centrally mediated alterations in BRS (Rong *et al*. 1999; Ouyang & Wang, 2000; Bechtold & Scheuer, 2006; Scheuer, 2010). In rats, administration of glucocorticoids in the rostral ventrolateral medulla (RVLM) (Rong *et al*. 1999) and the nucleus tractus solitarii (NTS) (Ouyang & Wang, 2000) rapidly alters the activity of baroreceptive neurons and depresses baroreflex control of HR. A cortisol‐induced impairment in BRS may be an important mechanism by which acute stress increases cardiovascular risk (Brotman *et al*. 2007); however, to our knowledge, there have been no prior studies assessing the direct acute effects of cortisol on resting cardiovagal BRS in humans. The effect of longer‐term oral glucocorticoid therapy on BRS in humans has been examined with equivocal results that are at odds with the animal studies noted above (Rong *et al*. 1999; Ouyang & Wang, 2000; Bechtold & Scheuer, 2006; Scheuer, 2010). Although 7 days of administration of hydrocortisone (200 mg day^−1^) increased BRS (Tam *et al*. 1997), no alteration in BRS was observed following 7 days of the synthetic glucocorticoid prednisone (60 mg day^−1^) (Cottin *et al*. 2015). Examination of the acute effects of glucocorticoids on BRS circumvents the potential confounding influence of longer‐term peripheral effects (e.g. change in total body water, sodium content, glucocorticoid receptor desensitization). Hydrocortisone acutely augments the diastolic blood pressure (BP) response to a cold pressor test (Heindl *et al*. 2006). However, whether this is attributable to an exaggerated peripheral vasoconstrictor response is unclear.

We aimed to determine the acute effects of hydrocortisone on: cardiovagal BRS, HRV, BP variability and cardiovascular reactivity to the cold pressor test and a mental arithmetic stress task. Cardiovagal BRS was principally evaluated using the modified Oxford pharmacological technique (sequential of bolus infusions of sodium nitroprusside and phenylephrine) but in the absence of an agreed ‘gold standard’ method of assessing BRS (Lipman *et al*. 2003; Malliani & Montano, 2004; Parati *et al*. 2004), ‘spontaneous’ approaches (sequence technique, transfer function analysis) were also utilized. Although the latter rely on spontaneously occurring fluctuations in heart period and BP to derive an index of closed‐loop cardiovagal BRS, the advantage of the former is that arterial pressure is moved through a wider range, thus perturbing the system to assess open‐loop cardiovagal BRS. We hypothesized that, in healthy young males, an iv bolus of hydrocortisone would acutely reduce cardiovagal BRS and HRV, as well as increase cardiovascular reactivity via increased vascular responsiveness.

## Methods

### Subjects

The study procedures were approved by National Research and Ethics Service Committee West Midlands – Edgbaston (11/WM/0298) and the study conformed with the standards set by the latest revision of the *Declaration of Helsinki*, except for registration in a database. Written informed consent was obtained from each participant. Ten male participants (median age 27.0 years, interquartile range 23.8–34.5 years, body mass index 24 ± 3 kg m^2^) were recruited from the University of Birmingham, UK, and surrounding areas. All participants were free from cardiovascular, pulmonary, renal, metabolic and neurological conditions, and none were taking any prescription or over‐the‐counter medications. Experiments were conducted in a quiet room, in standardized conditions with a temperature of 24°C. Participants were asked to abstain from alcohol consumption for 24 h prior to testing, as well as caffeine and food intake for 12 h prior to testing.

### Experimental protocol

Each participant was investigated on two occasions separated by 1 week, in accordance with a placebo‐controlled, single‐blinded, cross‐over design. Participants were randomized to receive either placebo or hydrocortisone on the morning of the first visit using a randomization method in two groups of five (Schulz & Grimes, 2002). The choice was blinded to the participant throughout the duration of the study but not the investigator. Computer generated random sequences were used to determine the order of drug administration. None of the subjects reported any side effects (e.g. allergic‐like reactions, nausea, abdominal pain and heartburn) and did not report being able to distinguish between hydrocortisone and placebo administration.

Participants attended the research laboratory at 09.00 h. An iv catheter was inserted into a superficial vein at the antecubital fossa for blood sampling and injections. Baseline blood samples were taken for biochemistry, including renal function (serum creatinine), electrolytes (sodium, potassium), plasma osmolality, baseline cortisol and adrenocorticotrophin hormone (ACTH). All participants were then administered either 200 mg of hydrocortisone (Solu‐Cortef; Pfizer, New York, NY, USA) or placebo (9% saline solution) iv. Following this, subjects were given a standardized light breakfast and observed in a quiet, temperature‐controlled room.

At 12.00 h, height and weight were measured and body mass index was determined (weight/height^2^). The participants assumed a comfortable supine position and were instrumented with the measurement equipment (HR, BP, leg blood flow). Further blood samples were taken (cortisol, ACTH, catecholamines, serum electrolytes, serum osmolality and haematocrit) after which participants rested quietly for a further 30 min. During the final 10 min of this period, data were recorded. Following this resting baseline, the following procedures were undertaken during which the participants remained supine: (i) sequential infusion of 100 μg of sodium nitroprusside (SNP) and 150 μg of phenylephrine (PE) 1 min later [modified Oxford Technique (MOT)] (Rudas *et al*. 1999); (ii) cold pressor test (immersion of right hand in a container of cold water at 4°C for 2 min) (Victor *et al*. 1987); and (iii) mental stress task [paced auditory serial arithmetic task (PASAT) for 6 min] (Gronwall, 1977). During the PASAT test, a series of single digit numbers was presented to the participants for 6 min using a pre‐recorded audio file on a computer. Participants were instructed to add each number that they heard to the previous number presented to them, and to retain the last number to add to the next number they heard (Gronwall, 1977; Veldhuijzen van Zanten *et al*. 2005). To make the task progressively more challenging, the numbers were presented every 3.5, 3.0 and 2.5 s, respectively, in three consecutive blocks each lasting 2 min. An experimenter checked their responses against the correct answers and alerted the participant with a loud buzzer noise with each incorrect answer, hesitation or once during every 10 additions if no mistakes were made. In addition, participants were instructed to view themselves in a mirror for the duration of the mental stress task to increase their levels of self‐awareness. Together, these elements of time pressure, social evaluation, punishment and self‐awareness have been shown to increase the provocativeness of the stress task (Veldhuijzen van Zanten *et al*. 2004). A 10‐point scale was used to obtain perceived pain and stress ratings from the subjects following the cold pressor test and the PASAT mental stress task, respectively.

Each test was separated by ∼20 min of recovery, such that the total duration of the autonomic function test battery was ∼60 min.

### Measurement of haemodynamic parameters

HR was measured using a lead II ECG (BioAmp; AD Instruments, Bella Vista, Australia). Beat‐to‐beat BP measurements were made using photoplethysmography (Portapres; Finapres Medical Systems, Amsterdam, The Netherlands) and calibrated with brachial BP measurements made using an automated sphygmomanometer (Omron 705IT; Omron Healthcare Europe BV, Hoopddorp, The Netherlands). Leg and forearm blood flow were measured simultaneously using venous occlusion strain gauge plethysmography (Joyner *et al*. 2001). In brief, a lightweight indium‐in‐silastic strain gauge was positioned around the right calf and the right forearm at the point of greatest circumference (Hokanson EC‐6 plethysmograph; DE Hokanson, Bellevue, WA, USA). Cuffs were placed around the wrist and ankle and inflated to a pressure of 200 mmHg and maintained for 1 min to achieve arterial occlusion (Burggraaf *et al*. 2000). Sixty seconds later, cuffs placed around the thigh and upper arm were rapidly inflated (within 0.75 s) to 50 mmHg (Hokanson E20 rapid cuff inflator and AG101 air source; DE Hokanson) to evoke venous occlusions. Venous occlusion was repeated three times during 1 min, with the forearm and thigh cuffs inflated for 5 s and then deflated for 10 s each time. Leg blood flow and leg vascular conductance (LVC) {[blood flow (mL 100 mL^−1^ min^−1^)/mean BP (mmHg)] × 1000)} were determined at rest, during the cold pressor test and during the PASAT mental stress task. Given the reported variability in the leg blood flow responses to mental stress (Carter *et al*. 2005), forearm blood flow and forearm vascular conductance (FVC) were also determined during the PASAT mental stress task. Ratings of perceived pain and stress were obtained using a 10‐point scale after the cold pressor test and PASAT, respectively.

### Blood sampling

Blood samples for analysis of hormones were centrifuged immediately and the plasma was stored at −80°C. Plasma ACTH levels were determined using an enzyme‐linked‐immunosorbent assay (ELISA) (Abnova, Taipei City, Taiwan). The sensitivity was < 1 pg mL^−1^ and the intra‐assay and inter‐assay coefficients of variation were ≤ 4.2% and ≤ 6.2%, respectively. Serum cortisol levels were determined using an ELISA (Abcam, Cambridge, UK). The sensitivity was 2.44 ng mL^−1^ and the intra‐assay and inter‐assay coefficients of variation were ≤ 9.0% and ≤ 9.8% respectively. Serum electrolyte concentrations, glucose, serum osmolality and haematocrit were determined in accordance with standard laboratory methods.

### Data analysis

Analogue signals were interfaced with an analogue‐to‐digital converter (PowerLab; AD Instruments) and a personal computer equipped with data acquisition software (LabChart; AD Instruments). Cardiovascular variables were sampled at a rate of 1000 Hz. Beat‐to‐beat values of HR and BP were calculated offline using LabChart software (LabChart, AD Instruments). BRS was assessed using three methods: the modified Oxford technique (*G*_MOT_) (Rudas *et al*. 1999; Adlan *et al*. 2017); the ‘sequence technique’ (*G*_SEQ_) (Parati *et al*. 1988); and low frequency transfer function gain (*G*_LFTF_) (deBoer *et al*. 1987). The modified Oxford technique involved sequential iv bolus infusions of sodium nitroprusside (100 μg) and phenylephrine (150 μg) 1 min apart, during which arterial BP and RR‐interval were simultaneously recorded (Rudas *et al*. 1999; Adlan *et al*. 2017). Infusion of these agents results in a fall and subsequent rise in arterial BP that activates and deactivates the arterial baroreceptors. The relationship between the arterial BP and RR‐interval was analysed during the phenylephrine‐induced rise in systolic BP. Analysis began at the first concordant change in systolic BP and RR‐interval after phenylephrine infusion until the systolic BP and RR‐interval changes were discordant (Rudas *et al*. 1999). Baroreflex delays were accounted for by associating systolic BP with concurrent (resting RR‐interval greater than 800 ms) and subsequent RR‐intervals (resting RR‐interval between 500 and 800 ms) (Pickering & Davies, 1973; Eckberg & Eckberg, 1982). RR‐interval values were averaged over 3 mmHg pressure bins to account for respiratory‐related variations (Ebert, 1990). Saturation and threshold regions of the baroreflex curve were excluded and the linear relationship between systolic BP and RR‐interval (*G*_MOT_) was determined using piecewise regression (Studinger *et al*. 2007). A minimum of 10 points was required and only values with *r* ^2^ ≥ 0.6 were accepted. The ‘sequence technique’ was used to provide a spontaneous measure of cardiac baroreflex sensitivity (Parati *et al*. 1988). Commercially available software (CardioSeries, version 2.4; CardioSeries, Ribeirão Preto, SP, Brazil) was used to identify sequences of three or more consecutive cardiac cycles where increases/decreases in systolic BP (of at least 1 mmHg) were related to lengthening/shortening in RR‐interval of the following cardiac cycle (i.e. lag +1). The slope of the regression line (RR‐interval *vs*. systolic BP) was calculated individually and all slopes were averaged to provide an overall sensitivity gain (*G*_SEQ_) (Parati *et al*. 1995). A minimum threshold of 1 mmHg for systolic BP and 1 ms for RR‐interval was applied, and only sequences with an *r* ^2^ > 0.8 were accepted. Low frequency transfer function gain (*G*_LFTF_) was determined using cross‐spectral analysis of systolic BP (input) and subsequent RR‐interval (output) in the low frequency range (0.047–0.156 Hz) (deBoer *et al*. 1987). Only values with a coherence ≥0.5 were included. The low frequency range has the advantage of representing spontaneous oscillations in BP and RR‐interval without the effects of breathing (La Rovere *et al*. 1998).

Time domain, frequency domain and non‐linear indices of short term HRV were determined from a 10 min resting period (Kubios HRV, Kuipio, Finland) in accordance with guidelines from the Task Force of the European Society of Cardiology and the North American Society of Pacing Electrophysiology (1996). Time domain indices included square root of the mean sum of the squares of difference between adjacent inter‐beat intervals (rMSSD), number of pairs of adjacent inter‐beat intervals differing by more than 50 ms in the entire recording expressed as an absolute value (NN50) and a percentage of all inter‐beat intervals (pNN50%). Frequency domain indices (using fast Fourier transform) of R‐R intervals included total power (TP), very low frequency power (VLF, range 0–0.04 Hz), low frequency power (LF, range 0.04–0.15 Hz) and high frequency (HF, range 0.15–0.4 Hz). Absolute values for TP, VLF, LF and HF were determined in addition to normalized units for LF, HF and ratio of LF/HF power. In addition, non‐linear indices of HRV were determined: SD of the Poincare plot SD1, SD2 and the SD1/SD2 ratio. rMSSD, NN50, pNN50% and heart rate fluctuations in the HF power range are suggested to be principally indicative of cardiac parasympathetic activity (Anonymous, 1996). The physiological correlates to LF power fluctuations and the LF/HF ratio remain uncertain (Eckberg, 1997). A Poincaré plot analysis was undertaken by plotting the values NNn+1 against the values of NNn (Sassi *et al*. 2015). SD1 provides an estimate for short term HR variability (Mourot *et al*. 2004), whereas SD2 is influenced by both parasympathetic and sympathetic activity (De Vito *et al*. 2002). The SD1/SD2 ratio provides a measure of the relationship between SD1 and SD2.

Time and frequency domain parameters of systolic and diastolic BP variability were determined from a 10 min resting period (CardioSeries). Time domain indices included SD and variation of coefficient (VC = SD/BP × 100). Frequency domain indices (using fast Fourier transform) included LF power (range 0‐0.04 Hz) and HF power (range 0.15–0.4 Hz). BP oscillations in the LF range are assumed to principally represent vasomotor activity whilst HF oscillations represent respiratory influences (Parati *et al*. 1995).

### Statistical analysis

Statistical analyses were performed using SPSS, version 19 (SPSS Inc, Chicago, IL, USA). Continuous variables were assessed for normality using the Kolmogorov–Smirnov test. Baseline differences in parameters were tested using a two‐tailed paired Student's *t* test. Non‐parametric data was log‐transformed (natural). The effect of treatment (with hydrocortisone or placebo) during each of the tasks was tested using repeated measures analysis of variance (ANOVA) with Bonferroni adjustments for multiple comparisons. Differences between the percentage changes (% Δ) in parameters from baseline during each cold pressor test and mental stress task were assessed using a two‐tailed paired Student's *t* test. For statistical purposes, when a measurement exceeded the range of assay detection, a value corresponding to the limit of detection was used. Based on a previous study (Heindl *et al*. 2006), a sample size calculation indicated that seven patients were required to show a mean difference in HR of 7 ± 5 beats min^−1^ before and after hydrocortisone. Data are expressed as the mean ± SD or geometric mean [95% confidence interval (CI)]. *P* ≤ 0.05 was considered statistically significant.

## Results

### Biochemical parameters

There was no significant difference in baseline concentrations of serum cortisol, ACTH, glucose, electrolytes (sodium, potassium), creatinine and plasma osmolality in the placebo and hydrocortisone trials (Table 1). Hydrocortisone elevated serum cortisol concentrations 3 h after infusion and suppressed ACTH compared to placebo. At 3 h post‐hydrocortisone administration, serum cortisol values exceeded the limit of detection in seven of 10 participants and a value of 1400 nmol L^−1^ was used for statistical purposes (i.e. the upper limit of detection). In the three participants for whom serum cortisol was measurable (as a result of a modified dilution strategy) at 3 h post‐hydrocortisone administration, serum cortisol was 2637 ± 42 nmol L^−1^. Hydrocortisone did not influence serum glucose, electrolytes, creatinine, plasma osmolality or haematocrit (hydrocortisone 42 ± 2 *vs*. placebo 41 ± 2%; *P* = 0.215).

**Table 1:** Effect of hydrocortisone on biochemical parameters

			ANOVA		
	Placebo	Hydrocortisone	Time	Drug	Interaction
Cortisol (nmol/L^−1^)					
09.00 h	132.9 (97.4–181.3)	154.5 (107.5–222.0)	<0.001	<0.001	<0.001
12.00 h	93.7 ± 37.0*	1771.2 ± 598.0*†			
ACTH (mmol/L^−1^)					
09.00 h	3.0 ± 0.7	3.6 ± 1.5	0.004	0.035	<0.001
12.00 h	3.4 ± 1.6	0.7 ± 0.4*†			
Glucose (mmol/L^−1^)					
09.00 h	4.5 (4.3–4.8)	4.3 (3.9–4.7)	0.325	0.772	0.158
12.00 h	4.3 ± 1.1	4.9 ± 1.1			
Na^+^ (mmol/L^−1^)					
09.00 h	141.3 ± 2.0	142.0 ± 1.6	0.623	0.799	0.079
12.00 h	141.8 ± 2.5	140.8 ± 1.1			
K^+^ (mmol/L^−1^)					
09.00 h	4.4 (4.1–4.6)	4.3 (4.2–4.5)	0.360	0.348	0.087
12.00 h	4.2 ± 0.2	4.4 ± 0.4			
Creatinine (μmol/L^−1^)					
09.00 h	83 (75–92)	82 (74–91)	0.062	0.003	0.207
12.00 h	79 (70–90)	76 (68–84)			
Plasma osmolality (mmol kg^−1^)					
09.00 h	291.4 ± 4.2	291.8 ± 3.2	0.589	0.833	0.564
12.00 h	291.9 ± 5.6	290.3 ± 6.6			

Values represent mean ± standard deviation or geometric mean (95% CI). Comparisons were made using a repeated measures one way analysis of variance (ANOVA) with Bonferroni correction. *Post hoc* significance *P* < 0.05 ^*^compared with 9 am, ^†^ compared with placebo. ACTH = adrenocorticotrophin hormone.

### Haemodynamic parameters

Resting HR was elevated following hydrocortisone administration compared to placebo (+7 ± 4 beats min^−1^; *P* < 0.001) (Table 2). Hydrocortisone also elevated resting systolic BP (+5 ± 5 mmHg; *P* = 0.008) but did not alter resting diastolic or mean BP, leg blood flow and LVC. Compared to placebo, there was a trend for increased forearm blood flow (*P* = 0.095) and FVC (*P* = 0.065) following hydrocortisone.

**Table 2:** Effect of hydrocortisone on resting haemodynamic parameters

	Placebo	Hydrocortisone	*t*	d.f.	*P* value
Heart rate (beats min^−1^)	50.9 ± 9.7	57.8 ± 9.0*	6.117	9	<0.001
Systolic BP (mmHg)	113.6 ± 7.9	118.8 ± 6.8*	3.395	9	0.008
Diastolic BP (mmHg)	64.7 (61.0–68.7)	64.3 (61.9–66.9)	−0.270	9	0.793
Mean BP (mmHg)	81.2 ± 5.8	82.5 ± 4.0	0.971	9	0.357
Leg blood flow (mL 100 mL^−1^ min^−1^)	1.9 ± 0.8	1.9 ± 0.7	−0.492	9	0.635
LVC (AU)	24.7 ± 12.3	22.6 ± 8.4	−0.023	9	0.982
Forearm blood flow (mL 100 mL^−1^ min^−1^)	2.4 ± 1.1	3.3 ± 1.2	1.866	9	0.095
Forearm vascular conductance (AU)	28.6 ± 13.7	39.8 ± 13.1	2.099	9	0.065

Values represent the mean ± SD or geometric mean (95% CI). Non‐parametric data were transformed and comparisons made using a paired *t* test. ^*^ *P* ≤ 0.05 compared with placebo. AU, arbitrary units.

### Cardiovagal BRS

**Figure 1:**
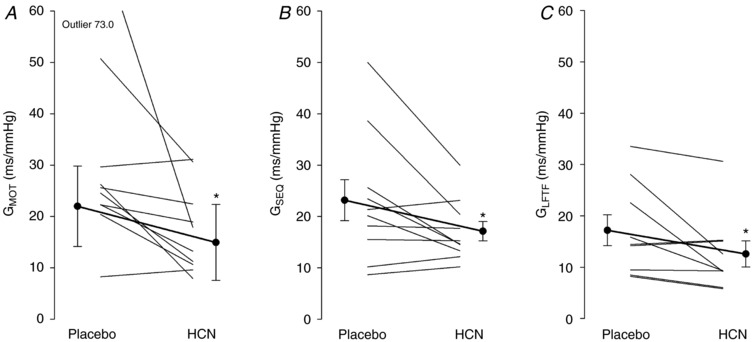
Cardiac baroreflex sensitivity Plot displaying cardiac baroreflex sensitivity following pre‐treatment with iv placebo and hydrocortisone (HCN). Baroreflex gain determined using the modified Oxford technique (*G*_MOT_) (*A*), sequence technique (*G*_SEQ_) (*B*) and low frequency transfer function (*G*_LFTF_) (*C*). Individual participants data are shown as thin lines. Bold lines indicate geometric mean (95% CI) (*A*) or group arithmetic mean ± SEM (*B* and *C*). ^*^ *P* ≤ 0.05.

Cardiac BRS was reduced with hydrocortisone administration (*G*_MOT_ geometric mean hydrocortisone 15.6, 95% CI = 11.1–22.1 *vs*. placebo 26.2, 17.4–39.5 ms/mmHg; *t* = −3.165, *P* = 0.011; *G*_SEQ_ 17.1 ± 5.9 *vs*. 23.2 ± 12.6 ms mmHg^−1^; *t* = −2.290, *P* = 0.048; *G*_LFTF_ 12.6 ± 7.6 *vs*. 17.2 ± 9.0 ms mmHg^−1^; *P* = 0.05) (Fig. 1). SNP and PE infusion induced a similar fall and rise in systolic BP in the hydrocortisone and placebo conditions (fall in systolic BP after SNP geometric mean 26, 95% CI = 21–34 *vs*. 26, 19–36 mmHg; *t* = 0.107, *P* = 0.917; rise in systolic BP after PE 26 ± 8 *vs*. 24 ± 11 mmHg; *t* = 0.628, *P* = 0.546).

### HRV

Time domain parameters of HRV (rMSSD, NN50, pNN50%) were reduced following hydrocortisone compared to placebo (Table 3). Absolute HF power was reduced following hydrocortisone, whereas there was a tendency for LF power to decrease (*P* = 0.057). There was no significant difference in normalized values of HF power, LF power and LF/HF ratio between trials. SD1 was reduced with hydrocortisone, whereas the numerical fall in SD2 was not statistically significant (*P* = 0.215). The ratio of SD1/SD2 fell following hydrocortisone administration (*P* = 0.042).

**Table 3:** Effect of hydrocortisone on resting HR variability and BP variability parameters

	Placebo	Hydrocortisone	*t*	d.f.	*P* value
HR variability					
rMSSD (ms)	84.1 ± 37.6	58.7 ± 28.8*	−3.801	9	0.004
NN50 (count)	242.2 ± 80.5	188.2 ± 100.7*	−2.491	9	0.034
pNN50 (%)	49.0 ± 16.7	33.8 ± 18.8*	−4.453	9	0.002
Total power (ms^2^)	5627 (3358–9429)	4258 (2757–6576)	−1.318	9	0.220
VLF power (ms^2^)	2089 (1210–3605)	2176 (1308–3620)	0.143	9	0.890
LF power (ms^2^)	1523 (816–2842)	982 (647–1491)	−2.180	9	0.057
HF power (ms^2^)	1587 (900–2800)	887 (502–1566)*	−3.192	9	0.011
LF power (nu)	49.0 ± 13.8	52.3 ± 13.8	0.711	9	0.495
HF power (nu)	51.0 ± 13.8	47.7 ± 13.8	−0.711	9	0.495
LF/HF ratio	1.12 ± 0.66	1.28 ± 0.71	0.640	9	0.538
SD1	59.6 ± 26.6	41.6 ± 20.4*	−3.801	9	0.004
SD2	105.0 (79.2–139.2)	90.1 (74.1–109.5)	−1.333	9	0.215
SD1/SD2	0.55 ± 0.18	0.44 ± 0.13*	−2.368	9	0.042
BP variability					
Systolic BP					
SD (mmHg)	6.4 ± 1.4	7.4 ± 1.2	1.997	9	0.077
VC (%)	5.5 (4.8–6.1)	6.3 (5.5–7.2)	1.731	9	0.117
LF power (ms^2^)	7.3 ± 4.7	7.4 ± 3.2	0.061	9	0.953
HF power (ms^2^)	1.5 (0.8–2.7)	1.4 (0.9–2.3)	−0.625	9	0.548
LF power (%)	29.9 ± 7.6	27.7 ± 5.9	−0.887	9	0.398
HF power (%)	12.2 ± 7.3	9.0 ± 5.4	−1.163	9	0.275
Diastolic BP					
SD (mmHg)	3.5 ± 0.8	3.1 ± 0.8	−1.337	9	0.214
VC (%)	5.4 ± 1.4	5.0 ± 1.4	−0.752	9	0.471
LF power (ms^2^ )	2.9 (1.9–4.4)	2.2 (1.6–3.1)	−1.472	9	0.175
HF power (ms^2^)	0.5 ± 0.2	0.5 ± 0.3	−0.079	9	0.939
LF power (%)	36.4 ± 6.6	36.9 ± 6.5	0.252	9	0.807
HF power (%)	6.7 ± 1.6	9.1 ± 4.1	1.776	9	0.110

Values represent the mean ± SD or geometric mean (95% CI). Non‐parametric data were transformed and comparisons made using a paired *t* test. ^*^ *P* ≤ 0.05 compared to placebo. HF, high frequency (0.15–0.4 Hz); LF, low frequency (0.04–0.15 Hz); NN50, number of pairs of adjacent NN intervals differing by more than 50 ms; pNN50, NN50 as a percentage of all NN intervals; nu, normalized units; rMSSD, root mean square of successive differences; SD, standard deviation of the Poincare plot; VLF, very low frequency (0–0.04 Hz); BP, blood pressure; VC, variation of coefficient (SD/BP × 100).

### BP variability

Time domain parameters of systolic BP variability tended to increase with hydrocortisone, whereas frequency domain parameters remained unaffected (Table 3). Hydrocortisone had no effect on diastolic BP variability.

### Cardiovascular reactivity

#### Cold pressor test

**Figure 2:**
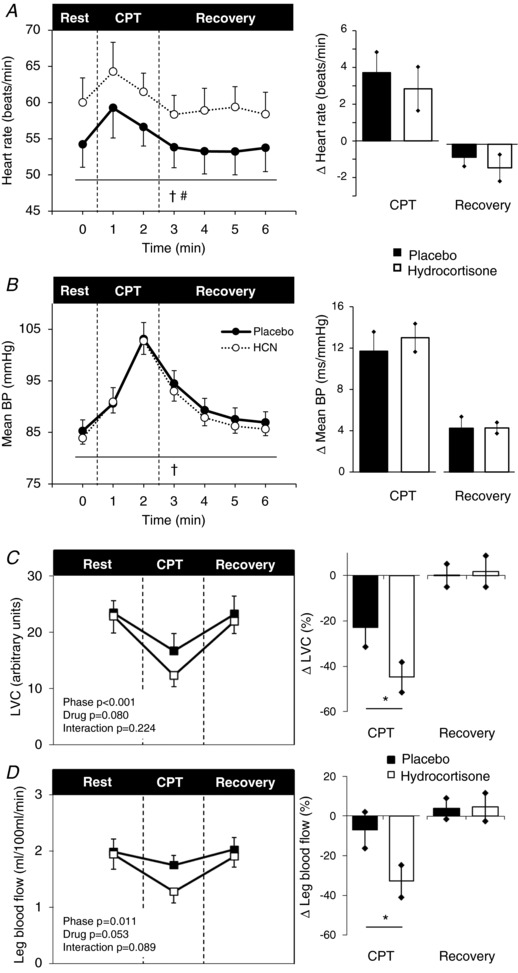
Cold pressor test Heart rate (*A*), mean BP (*B*), LVC (*C*) and leg blood flow (*D*) during rest, CPT and recovery following pre‐treatment with intravenous placebo (black) and hydrocortisone (white). Data represented as the group mean ± SEM. Times series is shown on the left. No significant interactions were found between time (rest, CPT and recovery) and drug (placebo or hydrocortisone) conditions when assessed using ANOVA with repeated measures. Bar charts on the right represent changes from baseline. Significance was determined using a paired Student's *t* test. ^*^ *P* ≤ 0.05 compared to baseline.

As expected, HR and BP rose during the cold pressor test, whereas leg blood flow and LVC fell (Fig. 2). Hydrocortisone administration resulted in a greater reduction in leg blood flow (–33 ± 24 *vs*. −7 ± 28 Δ%; *t* = 2.713; *P* = 0.027) and LVC (–45 ± 20 *vs*. −23 ± 26 Δ%; *t* = 2.798; *P* = 0.023) during the cold pressor test. Although there was a trend for increased systolic BP responses with hydrocortisone compared to placebo (+21 ± 10 *vs*. +14 ± 15 Δ mmHg; *t* = 1.640; *P* = 0.135), there were no differences in diastolic BP (+11 ± 4 *vs*. 10 ± 5 Δ mmHg; *t* = 1.562; *P* = 0.153) or HR (+3 ± 4 *vs*. 4 ± 4 Δ beats min^−1^; *t* = −0.657; *P* = 0.528) responses. There was no difference in the perceived pain rating during the cold pressor test between hydrocortisone and placebo (6.6 ± 2.2 *vs*. 7.1 ± 2.0; *t* = −0.785, *P* = 0.453).

#### PASAT mental stress task

As expected HR, BP, leg blood flow, forearm blood flow and FVC rose during the PASAT, whereas LVC tended to rise (Fig. 3). There was no difference in HR (+10 ± 8 *vs*. +11 ± 7 Δ beats min^−1^; *t* = −0.296; *P* = 0.774), systolic BP (+10 ± 13 *vs*. +11 ± 14 Δ mmHg; *t* = −0.279; *P* = 0.787), diastolic BP (+6 ± 6 *vs*. +6 ± 8 Δ mmHg; *t* = −0.098; *P* = 0.924), leg blood flow (+19 ± 20 *vs*. +25 ± 27 Δ%; *t* = −0.935; *P* = 0.185) and LVC (+10 ± 19 *vs*. +16 ± 25 Δ%; *t* = *vs*.0.934; *P* = 0.375) responses to the PASAT between hydrocortisone and placebo trials. However, there was a trend for reduced forearm blood flow (geometric mean +32, 95% CI = 14–69 *vs*. +56, 29–107 Δ%; *t* = −1.685; *P* = 0.126) and FVC (geometric mean +21, 95% CI = 8–59 *vs*. +50, 23–108 Δ%; *t* = −2.103; *P* = 0.069) responses following hydrocortisone administration. There was a tendency for reduced perceived stress (10‐point scale) during the PASAT with hydrocortisone (4.8 ± 1.9 *vs*. 5.7 ± 1.8 points out of 10; *t* = −0.785, *P* = 0.095); however, there was no difference in performance (geometric mean 74, 95% CI = 53–103 *vs*. 82, 74–91%; *t* = −0.828, *P* = 0.429).

Figure 3Mental stress taskHeart rate (*A*), mean BP (*B*), LVC (*C*), leg blood flow (*D*), forearm vascular conductance (*E*) and forearm blood flow (*F*) during rest, mental stress task (PASAT) and recovery following pre‐treatment with iv placebo (black) and hydrocortisone (white). Data represented as the group mean ± SEM. Times series is shown on the left. No significant interactions were found between time (rest, PASAT and recovery) and drug (placebo or hydrocortisone) conditions when assessed using ANOVA with repeated measures. Bar charts on the right represent changes from baseline. Significance was determined using a paired Student's *t* test. *P* ≤ 0.05 compared to baseline.

**Figure:**
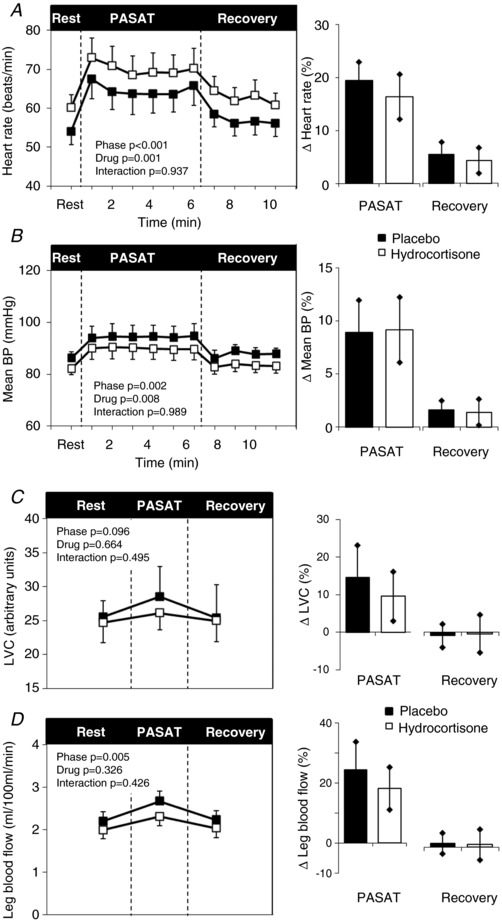

**Figure:**
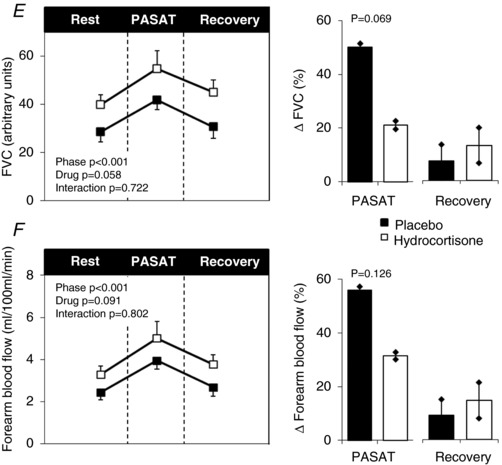

## Discussion

We report, for the first time, that acute hydrocortisone administration elicits an increase in HR that is associated with a reduction in cardiovagal BRS and HRV in young men. In addition, we observed that hydrocortisone administration significantly increased systolic BP and exaggerated the leg vasoconstrictor responses to cold pressor test, whereas vasodilatory forearm responses to mental stress tended to be reduced in young men.

### Cortisol and BRS

Reduced cardiovagal BRS and HRV are known to have deleterious cardiovascular consequences and are predictive of increased mortality (Wolf *et al*. 1978; Bigger *et al*. 1992; La Rovere *et al*. 1998; Mirizzi *et al*. 2013). There is an increased incidence of cardiac events during times of stressful life events (Rosengren *et al*. 2004). Mental stress evokes acute surges in cortisol and reduces BRS (Broadley *et al*. 2005; Durocher *et al*. 2011), with the latter being abolished by the inhibition of cortisol production with metatyrapone (Broadley *et al*. 2005). Similarly, intense physical stress (exercise) is also associated with decreased BRS, an increased risk of cardiac events, and a robust increase in cortisol (Jurimae *et al*. 2007; Fisher *et al*. 2015). Our observation that acute hydrocortisone administration reduced BRS in young men, may help explain the associations between acute stress‐induced increases in cortisol and cardiovascular risk (Brotman *et al*. 2007).

Our findings concur with animal studies showing that exogenous cortisol decreases cardiovagal BRS as a result of a modulatory influence within areas of the central nervous system important for baroreflex regulation (e.g. RVLM, NTS) (Rong *et al*. 1999; Ouyang & Wang, 2000; Scheuer & Bechtold, 2002; Bechtold & Scheuer, 2006; Scheuer, 2010). However, it is possible that cortisol acts upon other structures within the baroreflex arc such as afferent structures (e.g. vascular structures, afferent terminals), efferent target‐organ structures (e.g. sino‐atrial node or smooth muscle) and efferent nerves (Chapleau & Abboud, 2001). The cortisol‐induced increase in HR and systolic BP found in our study, as well as in previous studies (Takahashi *et al*. 1983; Dodt *et al*. 2000; Scheuer & Mifflin, 2001; Vozarova *et al*. 2003; Heindl *et al*. 2006), may suggest a centrally‐mediated resetting of the baroreflex. A rise in systolic BP would be expected to increase baroreceptor firing resulting in a compensatory fall in HR and inhibition of sympathetic outflow to the peripheral vasculature. Thus, it appears that an upwards and rightwards resetting of the baroreflex curve accompanies the observed reduction in cardiac BRS with acute hydrocortisone administration, as occurs in hypertension and heart failure. A reduction in cardiac parasympathetic activity may also explain the cortisol‐induced increased HR we report. We demonstrated that hydrocortisone acutely reduced several indices of HRV in young men that have been associated with cardiac parasympathetic activity (i.e., rMSSD, pNN50%, HF power) (Anonymous, 1996), although it should be recognized that the physiological correlates of HRV are debated (Parati *et al*. 2006; Taylor & Studinger, 2006). Nevertheless, reduced HRV is a pathogenic feature of cardiovascular diseases (e.g. hypertension and heart failure) and has been shown to be of prognostic significance after myocardial infarction (Wolf *et al*. 1978; Bigger *et al*. 1992; Anonymous, 1996). Rassias *et al*. (2011) demonstrated that endotoxin administration reduced HRV but, when preceded by a 6 h infusion of hydrocortisone (sufficient elevate plasma cortisol concentrations to ∼996–1380 nmol L^−1^) the day prior to receiving endotoxin, the reduction in HRV was blunted. Given the animal work demonstrating that cortisol acts within the central autonomic nuclei to reduce rather than increase cardiovagal control, a more probable explanation for the findings of Rassias *et al*. (2011) relates to a secondary effect on HRV resulting from the interaction between hydrocortisone and endotoxin. Indeed, administration of hydrocortisone in patients with sepsis reduces their requirement for vasopressors agents (Sprung *et al*. 2008) suggestive of an increase in vascular reactivity. Previous work in humans has provided mixed findings with regard to the effect of exogenously administered glucocorticoids on BRS, with both increases (Tam *et al*. 1997) and no differences (Cottin *et al*. 2015) being reported. The contrast with the present study findings maybe attributable to our selection of an acute iv dose (200 mg hydrocortisone), rather than a 1‐week oral dose (200 mg day^−1^ hydrocortisone (Tam *et al*. 1997) or 60 mg day^−1^ prednisone (Cottin *et al*. 2015), which obviates potential longer‐term peripheral effects (e.g. change in total body water, sodium content) with potential hemodynamic and neural autonomic control implications.

### Cortisol and cardiovascular reactivity

BP responses to the cold pressor test and mental stress have previously been used as an index of sympathetic reactivity of the vasculature (Freeman, 2006) and, if exaggerated, can predict the development of hypertension (Treiber *et al*. 2003). We found that cortisol acutely increased leg vasoconstrictor and systolic BP (trend) responses to cold water and inhibited forearm vasodilatory (trend) responses to mental stress in young men. Cortisol‐induced increases in pressor responsiveness have been demonstrated in prior studies with both acute (Heindl *et al*. 2006) and longer‐term (Sudhir *et al*. 1989) hydrocortisone supplementation and may reflect potentiation of catecholamines (epinephrine/norepinephrine) by cortisol in vascular smooth muscle (Besse & Bass, 1966). The altered vascular responsiveness following cortisol may also be explained by changes in endothelial function. Glucocorticoids reportedly alter endothelial function via stimulation and production of endothelin (circulating peptide with vasoconstrictor properties) (Morin *et al*. 1998) and inhibition of the nitric oxide synthase (NOS) isoforms inducible NOS and endothelial NOS (Radomski *et al*. 1990; Wallerath *et al*. 1999). In one study, cortisol inhibition blocked mental‐stress induced impairments in endothelial function as assessed by flow mediated dilatation (Broadley *et al*. 2005).

### Experimental considerations

It is a limitation of the present study that women were not recruited. Oestrogen can act at central autonomic nuclei (e.g. RVLM, NTS) to enhance BRS in rats (Saleh *et al*. 2000), although there is conflicting evidence regarding the influence of sex and ovarian hormones on cardiovagal BRS in humans (Fisher *et al*. 2012). The findings of the present study should only be considered applicable to men until additional studies are undertaken to clarify whether there are sex differences in the effect of acute hydrocortisone administration on cardiac autonomic regulation.

Our study design (single‐blinded, placebo‐controlled cross‐over) is a strength of the present study allowing a direct assessment of the effects of hydrocortisone. We cannot distinguish between the direct effects of cortisol and subsequent compensatory mechanisms (e.g. cortisol suppresses ACTH through negative feedback of the HPA axis). We therefore cannot rule out whether some of our findings are a result of ACTH suppression. The autonomic assessments used in the present study have inherent limitations because of the inaccessibility of the parasympathetic nervous system (e.g. HRV is used as an indirect measure of parasympathetic influences on the sinus node) and we did not measure sympathetic activity.

In the absence of a consensus regarding the criterion measure of BRS, we comprehensively evaluated BRS using the modified Oxford pharmacological technique (sequential of bolus infusions of sodium nitroprusside and phenylephrine) and ‘spontaneous’ approaches (sequence technique, transfer function analysis). The relative merits of methodical approaches used to assess BRS have been debated vigorously (Lipman *et al*. 2003; Malliani & Montano, 2004; Parati *et al*. 2004) and thus will only be covered briefly here. A key advantage to the ‘spontaneous’ approaches is that BRS can be non‐invasively derived from easily obtainable continuous recordings of heart period and BP without exogenous perturbation to the arterial baroreceptors. However, under these closed‐loop conditions, it is uncertain whether the heart period fluctuations are responding to, or contributing to, changes in BP (Diaz & Taylor, 2006). Such considerations are circumvented by the modified Oxford pharmacological technique where BP is briefly altered, thus allowing the assessment of open‐loop cardiovagal BRS. However, an important consideration of this technique is the potentially confounding influence of sodium nitroprusside, given the actions of nitric oxide on the sinoatrial node and autonomic nerves (Chowdhary & Townend, 1999; Hogan *et al*. 1999). Importantly, in the present study, the variety of methods that we used to assess BRS corroborated one another, with each indicating a decrease in BRS with acute hydrocortisone administration.

The hydrocortisone dosage used in the present study is clinically indicated in conditions such as severe inflammatory disease and adrenocortical insufficiency, where an iv bolus of up to 500 mg of hydrocortisone may be administered (Anonymous, 2018). Unfortunately, the measured cortisol concentration exceeded the limit of detection of the assay used in seven of the 10 participants. In the other three participants, cortisol concentration 3 h post‐hydrocortisone administration reached 2637 ± 42 nmol L^−1^. Therefore, we expect that cortisol concentration achieved was in excess of that previously reached in studies employing longer‐term 7‐day oral supplementation at the same 200 mg dose (Whitworth *et al*. 2005). The latter has been commonly used as a model of cortisol induced hypertension, whereas our approach may be more akin to a model of acute stress induced surge in cortisol, as may be seen evoked by robust stressors such as mental stress (∼550 nmol L^−1^) (Traustadottir *et al*. 2005; Jayasinghe *et al*. 2016), physical exercise (590 ± 81 nmol L^−1^, 30 min after a maximal 6000 m rowing ergometer test) (Jurimae *et al*. 2007), surgery (e.g. >1500 nmol L^−1^) (Lamberts *et al*. 1997) and severe illness (828–7173 nmol L^−1^) (Sandberg *et al*. 1956). Under basal conditions, cortisol is released in a pulsatile manner with a circadian and approximately hourly rhythm, resulting in an oscillating plasma concentration (Spiga *et al*. 2014). By contrast to a chronic supplementation of hydrocortisone where a desensitization of the glucocorticoid receptor may occur, the single iv bolus of hydrocortisone in that strategy we employed probably fully saturates all receptors and avoids issues of receptor desensitization. However, it is acknowledged that the plasma cortisol concentrations observed in the present study are towards the upper limits of the physiological range. Additional studies are required to determine the BRS and HRV responses to lower hydrocortisone doses, particularly in light of the observations of Rassias *et al*. (2011) who demonstrated that a moderate (sufficient to increase plasma cortisol to ∼996–1380 nmol L^−1^) but not high (sufficient to increase plasma cortisol to 2208–2760 nmol L^−1^) dose of hydrocortisone helped to maintain HRV during an endotoxin challenge in humans. The haemodynamic actions of glucocorticoids occur via multiple classical genomic and non‐genomic mechanisms (Falkenstein & Wehling, 2000) and the hydrocortisone dosing strategy used (i.e. acute *vs*. chronic) may influence the relative importance of these mechanisms. iv administered hydrocortisone (100 mg) reportedly produces a high peak concentration at 30 min before decreasing exponentially (Jung *et al*. 2014) and therefore may have declined throughout the autonomic function test battery. Only single measure of cortisol concentration was obtained following exogenous supplementation (3 h) and repeated assessments were not made. Therefore, it is unknown whether the endogenous cortisol responses to acute stress were altered when superimposed upon a background of exogenously administered cortisol; however, in the study by Heindl *et al*. (2006), no such interactive effects were noted.

During acute stress, there is complex co‐activation and interaction between the HPA axis and the sympathetic nervous system. The increased HR and systolic BP noted in the present study may represent heightened sympathetic activity, although this may not be the result of increased central sympathetic outflow *per se*. Glucocorticoids can up‐regulate cardiac β_1_‐adrenergic receptor sensitivity (Nishimura *et al*. 1997), which can potentiate the effects of circulating epinephrine and norepinephrine. In prior studies, resting muscle sympathetic nerve activity decreased (Dodt *et al*. 2000) or did not change (Vozarova *et al*. 2003; Heindl *et al*. 2006) following glucocorticoid administration. Peripheral vasoconstriction probably does not contribute to the rise in systolic BP because LVC remained unaffected in the present study and diastolic BP did not increase. The short time course of our study did not allow the renal actions of cortisol (i.e. increased sodium and water retention) to take effect and cannot explain the rise in systolic BP, especially because haematocrit and plasma osmolality were unchanged.

Our findings suggest a possible mechanism by which acute stress increases cardiovascular risk. During acute stress, the surge in cortisol may result in an acute depression in BRS and HRV, along with increased peripheral vasoconstrictor responsiveness, increased sympathetically‐mediated positive cardiac inotropic activity and impairment in vasodilatory mechanisms. Thus, this could provide a pro‐arrhythmic milieu that precipitates ventricular arrhythmia, myocardial infarction, left ventricular dysfunction and increased cardiovascular mortality.

## Additional information

### Competing interests

The authors declare that they have no competing interests.

### Author contributions

The experiments were performed in Department of Rheumatology, Dudley Group NHS Foundation Trust. AMA, JJCSVZ, GYHL, JFRP, GDK and JPF contributed to the conception, design and interpretation of data. AMA undertook the acquisition and analysis of data and, along with JPF, drafted the manuscript. All authors revised the work critically for important intellectual content, have approved the final version of the manuscript submitted for publication, and agree to be accountable for all aspects of the work in ensuring that questions related to the accuracy or integrity of any part of the work are appropriately investigated and resolved. All persons designated as authors qualify for authorship, and all those who qualify for authorship are listed.

### Funding

This study was supported by a grant from Arthritis Research UK (grant number 196633).
